# Access to maternal-child health and HIV services for women in North-Central Nigeria: A qualitative exploration of the male partner perspective

**DOI:** 10.1371/journal.pone.0243611

**Published:** 2020-12-10

**Authors:** Maryam Al-Mujtaba, Nadia A. Sam-Agudu, Nguavese Torbunde, Muktar H. Aliyu, Llewellyn J. Cornelius

**Affiliations:** 1 International Research Center of Excellence, Institute of Human Virology Nigeria, Abuja, Federal Capital Territory, Nigeria; 2 Division of Epidemiology and Prevention, Institute of Human Virology, University of Maryland School of Medicine, Baltimore, Maryland, United States of America; 3 Pediatric and Adolescent HIV Unit, Prevention, Care and Treatment Department, Institute of Human Virology Nigeria, Abuja, Federal Capital Territory, Nigeria; 4 Department of Health Policy and Vanderbilt Institute for Global Health, Vanderbilt University Medical Center, Nashville, Tennessee, United States of America; 5 School of Social Work and College of Public Health, University of Georgia Athens, Athens, Georgia, United States of America; USC Keck School of Medicine, Institute for Global Health, UNITED STATES

## Abstract

**Background:**

In much of sub-Saharan Africa, male partners play influential roles in women's access to maternal-child healthcare, including prevention of mother-to-child transmission of HIV services. We explored male partner perspectives on women’s access to maternal-child healthcare in North-Central Nigeria.

**Methods:**

Three focus groups were conducted with 30 men, purposefully-selected on the basis of being married, and rural or urban residence. Major themes explored were men’s maternal-child health knowledge, gender power dynamics in women’s access to healthcare, and peer support for pregnant and postpartum women. Data were manually analyzed using Grounded Theory, which involves constructing theories out of data collected, rather than applying pre-formed theories.

**Results:**

Mean participant age was 48.3 years, with 36.7% aged <40 years, 46.7% between 41 and 60 years, and 16.6% over 60 years old. Religious affiliation was self-reported; 60% of participants were Muslim and 40% were Christian. There was consensus on the acceptability of maternal-child health services and their importance for optimal maternal-infant outcomes. Citing underlying patriarchal norms, participants acknowledged that men had more influence in family health decision-making than women. However, positive interpersonal couple relationships were thought to facilitate equitable decision-making among couples. Financial constraints, male-unfriendly clinics and poor healthcare worker attitudes were major barriers to women’s access and male partner involvement. The provision of psychosocial and maternal peer support from trained women was deemed highly acceptable for both HIV-positive and HIV-negative women.

**Conclusions:**

Strategic engagement of community leaders, including traditional and religious leaders, is needed to address harmful norms and practices underlying gender inequity in health decision-making. Gender mainstreaming, where the needs and concerns of both men and women are considered, should be applied in maternal-child healthcare education and delivery. Clinic fee reductions or elimination can facilitate service access. Finally, professional organizations can do more to reinforce respectful maternity care among healthcare workers.

## Introduction

In many parts of sub-Saharan Africa, maternal and child health-related activities are considered almost exclusively women’s issues; however, men often serve as gateways for access to these health services [1–9]. Maternal-child health encompasses antenatal care as well as delivery and postpartum care, and includes integrated prevention of mother-to-child transmission of HIV (PMTCT) services [10]. The PMTCT cascade is a comprehensive package of services that includes maternal antenatal care, HIV testing, and initiation of antiretroviral therapy, and long-term postpartum health system engagement for HIV-positive women [11]. For HIV-exposed infants, the PMTCT cascade includes initiation of HIV prophylaxis, early infant diagnosis, initiation of antiretroviral therapy and follow-up care for HIV-infected infants, and final HIV testing for uninfected infants at 18 months of age [11].

Lack of, or poor access to maternal-child health services, including PMTCT care, has adverse effects on maternal and infant health outcomes, including higher risk of mother-to-child transmission of HIV [12–14]. In sub-Saharan Africa, the barriers to access and uptake of maternal-child health services include geographical and financial inaccessibility, poorly equipped facilities, unaffordable user fees, and clients’ perceptions or experiences of healthcare worker incompetence and/or unprofessional attitudes [15–20]. Socio-cultural factors relating to the traditional, expected roles of women in African societies also underlie many of these barriers [4, 17, 21–23]. In some of these settings, women are unable to independently make health-related decisions for themselves [9, 24], due to patriarchal norms [3, 25, 26]. However, men, as marital partners, brothers, fathers, and in other traditionally “masculine” roles, have the power to make family decisions, including the use of health services by women and children [3, 21, 27].

Heterosexual marriage is defined as living with a person of the opposite sex in a recognized union, and is highly prevalent in sub-Saharan Africa [28, 29]. Between 60% and 80% of women aged 15 to 49 years in sub-Saharan Africa are married [28], making the majority of women of childbearing age married, and subject to traditional marital roles and power dynamics. Monogamy (one man married to one woman) and polygyny (one man married to more than one woman), are the two most common forms of marriage in Africa, and both are strongly shaped by religious and traditional norms and practices [28, 30]. For instance, among Africans, Christians are more likely to practice monogamous marriage, while Muslims and traditionalists are more likely to practice polygyny [31]. Furthermore, relatively higher levels of education and urban residence are associated with monogamous unions among both men and women, while polygyny is associated with men’s advancement in age [30, 32].

Beyond impacting on patterns of marriage, factors such as education and age impact on male partners’ health decision-making. Furthermore, patriarchal norms often entrenched in, or reinforced by tradition and religion, tend to assign men sole/overriding financier, provider and decision-making roles, and this affects access to, and utilization of maternal-child health services by women [1–3, 5, 18, 33, 34]. These demographic, traditional, religious and interpersonal relationship factors synergistically interact to influence male partners’ decisions regarding maternal-child health [3, 5, 7, 34–36]. Of importance in making decisions are male partners’ level of knowledge and understanding of these services, their impact on maternal and child outcomes, and men’s motivation to participate in what is typically considered “women’s issues” [3, 5, 37, 38].

Support from male partners is especially important for pregnant women living with HIV, as they face additional challenges, including navigation of multiple PMTCT services, lifelong therapy, and HIV-related stigma and discrimination [39–41]. Male partner involvement in maternal health has been associated with significant improvements in PMTCT outcomes, including couple HIV status disclosure, maternal HIV testing, HIV treatment acceptance and adherence, and infant HIV-free survival [15, 41–47].

Poor male partner involvement has been reported among the major challenges facing Nigeria’s PMTCT program [10, 48]. This is important to explore and address, since Nigeria has had the second-largest number of pregnant women living with HIV every year for several years [49]. Among these women, antiretroviral therapy coverage is only 44% [49], and retention in PMTCT care is suboptimal [50, 51], contributing to an estimated 24,000 new pediatric HIV infections annually [52]. Furthermore, only 18% of HIV-exposed infants in Nigeria receive early diagnosis, and only 35% of HIV-infected children receive therapy [52]. These gaps provide an opportunity to better-characterize the multifactorial causes and devise contextually-relevant interventions. Male partner participation has been deemed important in PMTCT, thus, explorations of their views are important in finding solutions. In this paper, we present from the male perspective, factors influencing women’s access to maternal-child health and PMTCT services in North-Central Nigeria.

## Methods

### Study design and setting

This qualitative study was conducted between December 2012 and April 2013 in rural and urban communities of the Federal Capital Territory and Nasarawa State in North-Central Nigeria. In the Federal Capital Territory, antenatal HIV sero-prevalence for rural and urban communities were 5.0% and 6.1% respectively; corresponding data for Nasarawa were 9.0% and 5.0%, respectively [53]. Antenatal HIV sero-prevalence rates in these two states were much higher than the national average of 1.8% for rural and 3.2% for urban Nigeria [53].

This study was part of a formative assessment nested in the World Health Organization (WHO)-funded MoMent (Mother Mentor) Nigeria PMTCT implementation research study (37). The MoMent study evaluated whether structured peer support provided to women living with HIV by peer mentor mothers would improve postpartum maternal and infant PMTCT outcomes. The aim of the formative study was to gather contextual data (acceptability, facilitators and barriers) on the proposed intervention in particular, and on access to maternal-child health/PMTCT services in general, for the purpose of refining the intervention. To that end, we conducted 11 focus group discussions (FGDs) and 31 key informant interviews with various stakeholders, including healthcare workers, women living with HIV, and male partners from our study communities in North-Central Nigeria [54]. This paper presents and discusses data from three FGDs conducted with male partners only. Study methods and reporting were guided by the Consolidated Criteria for Reporting Qualitative Research (COREQ) [55]; a COREQ checklist is provided in S1 Appendix.

#### Study population and recruitment procedures

We recruited married or otherwise partnered men through purposive sampling based on their being married, and rural or urban residency. Participant selection was regardless of the men’s HIV status nor that of their female partners. We recruited participants at least 18 years old, targeting “older” men ≥40 years old and “younger” men 18 to <40 years old. Healthcare workers and community gatekeepers first approached eligible participants living in their catchment areas. Men who were interested were approached by the research team, briefed about the study, and given an FGD appointment date. Beyond recruitment activities, there was no established relationship with participants prior to study commencement. Recruitment was stopped once the target sample size of 10 men per group was reached.

On the appointed interview date, study staff sought written informed consent from all potential participants who presented. The informed consent process and form were individually explained to potential participants, and questions addressed in English, Pidgin English or Hausa (the dominant local language) according to their preferences. Thereafter, individuals who agreed to participate signed individual consent forms; witnessed thumbprints were obtained from those who could not read or write. All participants received refreshments and reimbursements corresponding to transport costs applicable to the day of the focus group only. The three FGDs were conducted at two primary healthcare centers (one semi-rural and one rural), and in one urban community.

#### Data collection

Prior to each discussion, a 15-30-minute interviewer-administered survey was used to capture participants’ socio-demographic data, including age, residence (rural or urban) religious affiliation, marital and educational status, occupation, number of children, and history of HIV testing. All sessions were conducted as dual-moderator focus groups [56] where a second facilitator ensured that questions and probes posed by the primary facilitator were understood and answered by participants. Discussions were guided by a semi-structured questionnaire (S2 Appendix) and conducted by a trained facilitator and co-facilitator who were fluent in English, pidgin English, and/or Hausa. An observer took notes on non-verbal cues during these discussions; these field notes were used to assist in data interpretation.

The questionnaire first explored participants’ health-seeking behavior, views on maternal-child health service delivery, and their perceived responsibilities, expectations, and involvement in these services as male partners. The discussion then focused on their knowledge of maternal-child health, views on HIV and associated stigma, PMTCT, and peer support for maternal health in general and PMTCT in particular. Lastly, the discussion explored participants’ views on access to maternal-child health and PMTCT services for women as wives and life partners. All FGDs took 1 ½ to 2 hours to complete and were audio-recorded in private rooms either at healthcare facilities or at community venues agreeable to participants. Besides consented participants, facilitators and observers, no other persons were present during the focus groups.

### Transcription and data analysis

The multilingual staff who facilitated the FGDs also transcribed the recordings and contributed to the analysis. All FGDs were transcribed verbatim in English, or translated from pidgin English or Hausa to English where necessary. Personal identifiers were removed from the transcripts. We adopted a Grounded Theory approach to underpin our qualitative study, where we aimed to build theory from the data collected, rather than apply preformed theoretical assumptions to the data [57]. Given that the views of male partners on maternal health services had not yet been documented in this study setting, the research team was uninformed about the type of answers that participants were likely to provide. Thus, a Grounded Theory approach was deemed most appropriate.

In this inductive approach, we applied thematic analysis methodology after collecting data from all three focus groups as follows [58]: each of the three FGD transcripts underwent manual initial coding by a pair of analysts, for a total of six analysts (four men (including LJC) and two women (MAM and NASA)). In initial coding, analysts read and hand-coded their assigned transcripts line by line, tagging recurring words and phrases as codes. After re-reading and further coding of transcripts, similar inductive codes were then collated according to identified patterns. The data-derived inductive codes related to participants’ perspectives with respect to access to maternal-child health services for women, norms contributing to, or limiting access to services, and the psychosocial and other support that women needed. These codes were categorized in hierarchical fashion into a coding tree, with parent (main) codes (“access”, “norms”, “support”) under which related subcategory codes were arranged [59]. Ultimately, categories were combined or expanded to represent emergent themes. The grouping of codes, determination of categories, and reviewing, defining and naming of emergent themes were done by group consensus at meetings where all analysts were present.

### Ethical approvals

The study was approved by the Nigerian National Health Research Ethics Committee, the Ethics Review Committee of the WHO, and the Institutional Review Boards of the University of Maryland Baltimore, and the University of Georgia Athens.

## Results

We interviewed 30 married men in three focus groups. Overall, mean age was 48.3 (±13.4) years; 11 men (36.7%) were ≤40 years old, 14 (46.7%) were 41 to 60 years old, and five (16.6%) were >60 years. Twenty-four (80.0%) participants lived in rural communities, even though some of them were recruited in an urban location (Table 1). Two FGDs constituted predominantly of Muslims (mean ages 56.2 and 47.4 years), while the third group was exclusively Christian (mean age 41.4 years). Overall, 60% of participants reported being Muslim and 40% Christian, with no other religious affiliation nor “no religion” declared. Over half of the participants (17/30, 56.6%) reported being in polygynous unions, and a majority (19/30, 63.3%) had five or more living children. Nearly 60% (17/30) of the men interviewed reported never being tested for HIV.

**Table 1:** Socio-demographic characteristics of study participants.

	Group 1	Group 2	Group 3	All Groups
Sample Size	N = 10	N = 10	N = 10	N = 30
**Age, years: mean (SD)**	56.2 (± 17.3)	47.4 (± 9.7)	41.4 (± 8.2)	48.3 (± 13.4)
**Other Characteristics: n (%)**				
**Residence**				
Rural	10 (100.0)	10 (100.0)	4 (40.0)	24 (80.0)
Urban	0 (0.0)	0 (0.0)	6 (60.0)	6 (20.0)
**Religious Affiliation**				
Christianity	1 (0.0)	1 (0.0)	10 (80.0)	12 (40.0)
Islam	9 (100.0)	9 (100.0)	0 (0.0)	18 (60.0)
Other religion	0 (0.0)	0 (0.0)	0 (0.0)	0 (0.0)
No religion	0 (0.0)	0 (0.0)	0 (0.0)	0 (0.0)
**Type of Union**				
Monogamous	1 (10.0)	2 (20.0)	9 (100.0)	12 (40.0)
Polygynous	9 (90.0)	8 (80.0)	1 (10.0)	18 (60.0) ^a^
**Number of living children**				
None	0 (0.0)	0 (0.0)	2 (20.0)	2 (6.7)
1–2	1 (10.0)	1 (10.0)	1 (10.0)	3 (10.0)
3–4	0 (0.0)	1 (10.0)	5 (10.0)	6 (20.0)
5+	9 (90.0)	8 (80.0)	2 (20.0)	19 (63.3)
**Formal Education**				
None	3 (30.0)	4 (40.0)	0 (0.0)	7 (23.3)
Primary	3 (30.0)	0 (0.0)	2 (20.0)	5 (16.7)
Secondary	0 (0.0)	4 (40.0)	5 (50.0)	9 (30.0)
Post-secondary +	4 (40.0)	2 (20.0)	3 (30.0)	9 (30.0)
**Community Leadership****^b^**				
Yes	10 (100.0)	9 (90.0)	5 (50.0)	24 (80.0)
**Current Main Occupation**				
Unemployed	0 (0.0)	1 (10.0)	0 (0.0)	1 (3.3)
Civil servant	0 (0.0)	1 (10.0)	0 (0.0)	1 (3.3)
Business/Commercial	0 (0.0)	1 (10.0)	0 (0.0)	1 (3.3)
Professional^c^	1 (10.0)	3 (30.0)	10 (100.0)	14 (46.7)
Farmer	8 (80.0)	3 (30.0)	0 (0.0)	11 (36.7)
Artisan/Handyman	1 (10.0)	1 (10.0)	0 (0.0)	2 (6.7)
**Ever Tested for HIV**				
Yes	1 (10.0)	5 (50.0)	7 (70.0)	13 (43.3)
No	9 (90.0)	5 (50.0)	3 (30.0)	17 (56.6)

SD: standard deviation.

^a^Includes 13 men with two wives; 3 men with three wives, and 2 men with four wives.

^b^Includes teachers, religious leaders, and traditional rulers.

^c^Includes teachers and commercial drivers.

### Focus group findings

**Fig 1:**
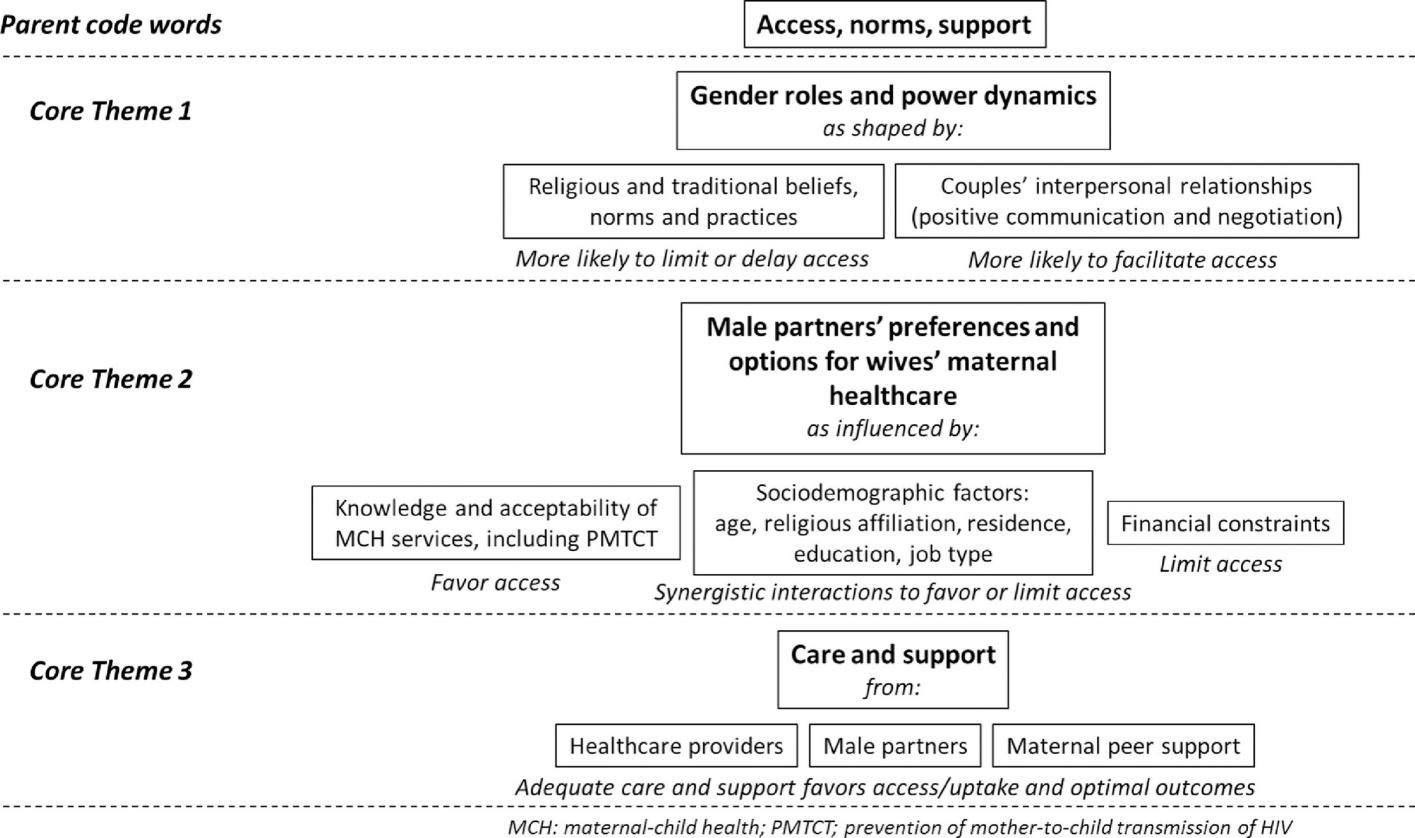
Core themes in women’s access to maternal-child health services from the male partner perspective.

With regards to access to maternal-child health and PMTCT services for women, three core themes emerged out of the focus groups: gender-power dynamics; male partners’ individual knowledge, acceptability and preferences as influenced by factors such as financial circumstances; and the availability of ancillary psychosocial and maternal support for pregnant and postpartum women. Fig 1 presents these themes and their interrelationships, as deciphered from our thematic analysis. The figure also presents major factors shaping these themes, such as male partner religion, type of job, age, education, and place of residence.

#### Gender-power dynamics and women’s access to maternal-child health services

Some nuanced inter-group differences emerged with regards to women’s access to maternal-child health services. Muslim-predominant groups (G1 and G2) expressed the expectation for women to seek permission before accessing services:

‘*She ought to ask for permission before going to the clinic because she is under my authority*. *But I do give her permission to go’*–G1 participant 1.

*‘There are rules; husbands are usually supposed to provide permission for our wives to go for medical care*.*’*-G2 participant 1.

However, these groups were also in agreement that in emergency situations when the male partner was not available, women were allowed to seek maternal care on their own.

*‘But if an emergency happens*, *they are allowed to go to hospital in your absence and then they can call you and inform you*.*’–*G2 participant 1.

Consensus from the Christian group (Group 3) was that women did not necessarily need to seek male partner permission to access services, but rather provide information:

*If the husband and wife understand the reasons or benefit or advantages of going to the hospital*, *there is no need calling for permission*, *rather you call it consent*. *Not that you are taking permission*, *but you just want to let him be in the know that you are going to the hospital*.*–*G3 participant 3.

However, a few Christian participants found religious relevance in a woman seeking approval to access care:

*‘Biblically*, *the woman is supposed to tell him…You and your wife are one*, *so if anything happens to her*, *it happens to you as well*.*’–*G3 participant 2.

There were others who relied on interpersonally-defined dynamics with their wives to make such decisions:

*‘As for me and my wife*, *people call us twins*. *There is love between us*. *We decide and do things together*.*’-*G3 participant 1.

#### Knowledge, acceptability and other male partner factors affecting women’s access to maternal-child health services

Beyond religious practice, participants’ preferences for their female partners’ care was influenced by their knowledge and acceptability of maternal-child health services as well as factors such as age, rural versus urban residence, finances, and employment.

*Knowledge of maternal-child health/PMTCT services*. Participants expressed varied levels of knowledge on the schedules and components of facility-based maternal care, especially antenatal care.

*‘It depends*. *Sometimes they will go about 7 times or more*, *or less than 7*. *It depends on how the woman is doing or if the doctor asks her to go for tests or something of that nature*.*’*–G2 participant 1.

‘*Initially when they get to the hospital*, *they have to do the pregnancy test*, *then she does the blood tests*, *HIV test and other varieties of tests*, *including malaria test*. *As a pregnant woman*, *she could be vulnerable to other diseases*, *which if not well taken care of*, *will affect the baby and anything could happen*.’-G3 participant 2.

Respondents also expressed some knowledge on what PMTCT services entailed:

*‘I believe when a woman tests positive to HIV during pregnancy*, *I think the specialist will administer some drugs to the woman that will reduce the virus in her… before she delivers the baby’–*G1 participant 2.

*Acceptability of*, *and access to formal healthcare services*. There was consensus that formal facility-based care was preferable, especially for women living with HIV and those with other complications:

*‘It is not bad to deliver at home if there is no problem*, *but it is preferable for them to deliver in the hospital*.*’–*G1 participant 2.

*‘They* (healthcare workers) *know what to expect in a woman and the kind of treatment to administer…*, *and the baby in the womb will also be okay*. *The traditionalist we have at home will not be able to have such experience*, *so it is quite good to go to the hospital so you can get such special treatment*.*’–*G3 participant 5.

However, participants expressed concern that access to facility-based services could be limited due to financial barriers:

‘*One of the things we encounter when going to the hospital is having to pay for some services*, *and if the fees are high*, *some of us would not be able to come to the hospital because of that*’–G2 participant 1.

*‘Private clinics distance at times*, *consumes a lot of our money more than anything*.*’* -G2 participant 3.

Male partners’ occupation could also affect the family/women’s access to care:

*‘‘Since our (commercial driver) jobs require us to travel a lot*, *there may be times where we are unavailable or unreachable by phone*.*’–*G3 participant 1.

*‘To many of us farmers*, *health and food are more important than money*. *When God gives you food to eat and you are in perfect health*, *the money will definitely come someday*.*’-* G2 participant 1 (with affirmation from majority of group.)

Generational/age influences also affected uptake:

*‘Before she became my wife*, *she patronized herbal medicine*, *because her father favoured herbal medicine*, *but since she became my wife*, *we patronize the hospital*.*’* G3 participant 2.

Rural versus urban residence also influenced utilization of services, especially due to poor availability and infrastructure:

*‘…healthcare amenities are beneficial to people in the urban areas and not for rural area dwellers…For us in this community*, *government has not been trying their best at all*, *because if we have enough facilities*, *we shouldn’t be rushing to get treatment elsewhere*.’ G2 participant 2.

*Healthcare worker preference in terms of gender*. There were mixed responses across the Muslim-predominant groups on this topic. G2 respondents mostly preferred their wives to be attended to by female healthcare workers because of religious practices:

‘*Islam says that women should attend to women*. *Even in death*, *when they are preparing the body*, *a woman should take care of the woman’s body*.*’–*G2 participant 10.

However, G1 respondents did not express any healthcare worker gender preferences:

*‘As for me*, *it doesn’t mean anything to me*. *In the process of looking for a female doctor*, *something might go wrong*. *A male doctor delivered my two children*. *So for me*, *the gender of the doctor or midwife doesn’t count*.*’–*G1 participant 8.

The Christian-predominant group had no strong preferences:

*‘So we know that in the hospital of today*, *we have the quack and the professional doctors*. *Whosoever has the experience and knowledge should do it*, *irrespective of man or woman*.*’–*G3 participant 2.

#### Maternal-child health services and support for women

*Healthcare worker attitudes toward women*. Rude healthcare workers were considered a deterrent for male partners to encourage their wives to access MCH services:

‘*Sometimes*, *what makes me not send my wife to the hospital is that some of the nurses are too harsh on the woman*, *either beating or insulting her*.’- G3 participant 1.

Some men observed that accompanying their wives to clinic would draw better treatment from healthcare workers:

*‘There is no respect from the nurses to our wives except if they are accompanied to the clinic by the husbands*. *There is some kind of preferential treatment that is given to your wife while you are in the clinic with her*, *and there are insults that she faces when you are not there with her*, *from these same nurses*.*’—*G3 participant 4.

*Support from male partners*. Some participants described attending clinic with their wives:

*‘I accompany my wife to the clinic because when a woman tells you she is pregnant*, *it is a thing of joy*, *and if you want to show love*, *I believe attending the clinic with her for one or two hours is not too much*.*—*G1 participant 5.

Some respondents dropped off or accompanied their wives to clinic, but did not attend appointments with them. This was due to the notion of maternal-child health services as “women’s issues”, and also for lack of time.

*‘I don’t usually enter the clinic because it is like ‘women’s affairs’*, *it is as if I will be intruding if I go inside*.*’—*G1 participant 10.

‘*Because of mostly time factors*, *I mostly just drop her at the ANC (antenatal care) clinic*.*’—*G3 participant 2.

Furthermore, healthcare workers could also be hostile to male partners:

‘*For the delivery of my last son*, *the midwife harassed me on the reason why I came with my wife*. *I told her it was my right to come with her to the clinic*. *She told me I wouldn’t be able to sit down close to my wife*, *and then she dragged me away*.*’*—G1 participant 1.

*Support from other women and peers*. Overall, respondents demonstrated enthusiastic acceptability of support from other women, particularly trained peer support (also known as Mentor Mothers or Expert Mothers) for pregnant and postpartum women, including those living with HIV:

**‘***You see*, *why I said YES in capital letters is this*: *the services these ladies are going to provide here are similar to what our mothers would have been doing for our wives if we were all living together*. *This one is more advantageous because the women are trained*.*’–*G3 participant 1.

‘The HIV-positive woman needs to go to the hospital frequently for her medicine after birth, and so might need more help. She should also not be isolated.’

*-* G1 participant 7.

## Discussion

Our findings indicate that in our study setting, male partners have, and are aware of their considerable influence on women’s access to maternal-child health and PMTCT services. Several factors play into this influence, particularly religious and traditional norms of gender roles, male partner maternal-child health knowledge, formal education level, rural versus urban residence, financial status, type of employment, and healthcare worker attitudes.

Our results support studies from Nigeria [18, 34, 60, 61] and other African countries [7, 21, 62, 63]. However, it is evident from our findings and others that these roles are often entrenched in patriarchal mores reinforced by tradition and religion [21, 30, 31, 35]. Men are customarily regarded and empowered as major decision-makers and financiers who may facilitate, but are not to participate in maternal-child healthcare, and women are to comply with measures found acceptable by male partners and/or the family/community to ensure the birth of a healthy baby [21, 64–67]. As a result, women who would otherwise access care may be unable to do so if their male partner is in disagreement, and men who desire to actively participate in maternal-child healthcare may be derided and stigmatized as non-masculine.

Gender inequality in both monogamous and polygynous unions has been reported as a major barrier to quality health service access for women in different African countries [22, 38, 68–71]. Women’s access to care and male engagement in the context of monogamous versus polygynous unions did not feature strongly in our findings. However men who identified as Muslim were older, more likely to be polygynous and rural-dwelling, and expressed stronger patriarchal opinions about women’s health-decision-making and access to care. Younger age and urban residence have been associated with better birth preparedness and engagement among male partners in African countries [38, 72, 73]. Besides being younger, the Christian-predominant focus group had more exposure to formal education. Thus, education and age, rather than religious affiliation, may have influenced their more egalitarian views about women’s decision-making in accessing healthcare. Additionally, while 40% of them lived in rural areas, these Christian participants had occupations that required frequent travel into urban areas e.g., long-distance commercial driving. Thus, they may have had more exposure to cosmopolitan lifestyles and media messages and were likely better-adapted to changing gender roles.

Strategies addressing gender inequality in health aim to increase women’s agency, i.e., their ability to make choices and decisions about their own health, and to act upon them [74]. Gender mainstreaming requires that concerns and implications for both men and women are considered in policy-making and program implementation [74]. The WHO recommends integration of gender mainstreaming into health policy and practice to catalyze gender equity. However, this cannot be achieved without the engagement of religious, traditional and other community leadership, as well as working with men and boys to transform harmful patriarchal norms, behaviors, and practices [47, 74].

Some of our participants demonstrated some knowledge of antenatal care and PMTCT services, however in the absence of quantitative assessments, we cannot make concrete conclusions on their level of knowledge. Nearly a quarter of our participants had no formal education, however, 60% had attained secondary or higher levels of education. Lack of adequate male partner knowledge on maternal-child health may lead to poor access and/or inadequate care, and consequently higher rates of maternal-infant morbidity and mortality [7, 9, 22, 36, 47]. Other studies in Nigeria, Ethiopia and Tanzania report that among male partners, birth preparedness and complication readiness was overall low, but positively correlated with being young, married, completing tertiary-level education, accompanying wives to antenatal care, and urban residence [38, 72, 73]. In PMTCT, HIV knowledge among male partners is an enabler of their participation [47]. Maternal-child health programs can meaningfully engage men of different health knowledge/formal education backgrounds to promote “education by participation”, to improve the breadth and quality of their knowledge. This includes male peer education/support, engaging healthcare workers and community leaders to drive male participation, and routinized couples’ HIV testing, disclosure and counselling services [47, 75].

In our study, 43% of 30 male partners interviewed reported never having tested for HIV. Without targeted interventions, proportions of men not/never tested for HIV are reported as approximately 97% in Nigeria [48, 76] and between 35% and 95% in other African countries [77]. In addition to HIV knowledge, prior HIV testing facilitates male partner involvement in, and women’s access to PMTCT services [47]. Strategies to increase testing uptake among male partners include couples-, mobile-, home-based- and self-testing integrated into facility-based maternal-child health services and both facility and community HIV prevention programs [47, 77, 78].

Our findings indicate that male partners found financial barriers particularly problematic in their wives’ access to healthcare services. These included unaffordable, often-hidden clinic user fees, and transportation costs to access adequate services, especially for rural residents. As in other countries, poor health infrastructure and geographical inaccessibility of clinics is encountered disproportionally by rural Nigerian residents, and this compounds their healthcare costs [68, 79]. Distance from health facility, facility and transportation infrastructure and fees are critical factors in health service access [75, 80, 81]. Health systems and policy should address full implementation of subsidized costs to facilitate access and uptake; doing so will facilitate gender and socioeconomic equity, especially as pertains to accessing healthcare [80].

Discourteous attitudes of healthcare workers, particularly towards pregnant and postpartum women, are well-known barriers to access [16, 34, 82]. Similar to findings in other African countries [5, 7, 9, 21, 83–86], poor healthcare worker attitudes discouraged our participants from accompanying their wives to health facilities. Healthcare worker hostility was cited as the most common reason for male partners’ absence at delivery for pregnant women in North-Central Nigeria [87]. Strategies to accommodate male partners are important for their engagement and positive impact in maternal-child health [47, 65, 66]. These include making space and time for men’s health services and male peer support [47, 88]. The social stigma of men being in “women’s spaces” needs to be addressed by both communities and health systems, which should also ensure that these spaces are more male-friendly [7, 36, 62]. The evidence on strategies to promote respectful maternity care among healthcare workers in Africa are scarce, and impact may be short-lived [89, 90]. The WHO however recommends health policy-, organizational/health system- and community-level approaches to respectful maternity care for women and their families, recognizing the role of professional societies in pre-service training and licensing requirements, and a rights-based approach in making sustainable change [91].

Poor healthcare worker attitudes notwithstanding, it is interesting to find that men in our study reported observing better treatment for their wives when couples attended clinic together. Studies have reported fast-tracking of services for women who attend antenatal clinics with their male partners, however, there is also withholding of services, reprimands or even fines for women who attend without their partners [21, 47, 92]. Some of these strategies may have unintended negative consequences, and also discriminate against women who do not have a living or otherwise available or involved male partner. Women should not be punished for the inability or failure of their male partners to participate in maternal-child health care [85].

Consistent with our prior findings among women and other stakeholders in this study setting [34, 93], male partners found maternal peer support agreeable and necessary, regardless of the women’s HIV status. Lay peer support is an effective, acceptable and scalable intervention, particularly for maternal and infant PMTCT outcomes in sub-Saharan Africa [50, 94–98]. Mentor mothers can facilitate engagement of male partners in PMTCT; it is policy in Kenya’s national PMTCT program [99]. These women can be further engaged to provide education, screen for and initiate case management for intimate partner violence among their clients [100–102]. Besides providing health education and assistance to navigate services, mentor mothers can provide moral support and education to other women, specifically on increasing their agency and recruiting male partners to become better involved in maternal-child health. Mentor mothers’ acceptability among male partners thus strengthens the case for policymakers and implementers to scale up this intervention in PMTCT programs.

Much of our analytical findings fit within the Social Role Theory [103], which focuses on the perceptions of the roles people play in society. In this study, it speaks to gender roles, where male partners have specific, albeit stereotypical beliefs and expectations about their roles as men, the roles of women, and how they relate to each other in the context of maternal-child health. Given the complexity of Nigerian society relating to gender, religion, language and ethnic background, the narratives presented here were based on long-standing role expectations that are far older than the HIV/AIDS epidemic. These role expectations continue to influence whether and how male partners engage the health system for themselves and on behalf of their wives. As such, gender roles will continue to play an important part in the development of gender-based interventions in Nigeria. The Grounded Theory approach helps us to place the elicited issues and themes relating to healthcare access in the larger socio-cultural context of gender roles for Nigerian men and women.

### Study limitations

Our study is not without limitations. First, the three focus groups described here were a subset of 11 FGDs and 31 key informant interviews conducted as part of a formative study. While we may have reached thematic saturation for the larger study, we may not have achieved it for male partner-specific viewpoints. Second, the majority (80%) of participants described themselves as community leaders. While their opinions may reflect prevailing sentiments in their communities, as leader-representatives, social desirability bias could have played into their responses. Third, participants in the Christian group were younger than those in the two Muslim-predominant groups; differences in opinion may have been due to age and not necessarily or solely due to religious affiliation. Furthermore, place of residence or recruitment may have influenced opinions: the Muslim-predominant groups were recruited largely from rural areas, while most of the Christian group were urban residents. Lastly, while we explored maternal-child health knowledge among male partners, we did not conduct objective knowledge assessments to further buttress our points. A mixed-method study with a larger, more representative sample may have provided quantitative data to complement the qualitative findings.

## Conclusions

Male partners play decision-maker, financial provider and support roles for women in their utilization of maternal-child health services in our Nigerian study setting. These roles, and whether they are ultimately supportive of women or not, are synergistically influenced by multiple factors, including traditional and religious norms, and male partners’ age, level of education, socioeconomic status, and rural or urban residence. Gender mainstreaming with community stakeholder engagement that includes women can reduce harmful gender norms and advance gender equity in women’s healthcare access, ultimately improving maternal-infant outcomes. Robust and sustained health system changes that mitigate cost, transport and healthcare worker-associated barriers need attention. Objective measures of male partner knowledge regarding maternal-child health and PMTCT are needed, as well as data on how different types of marital union influence access and outcomes in maternal-child health. Finally, we need best practices for how lay peer support for both men and women can be better-leveraged to improve male partner engagement and optimal outcomes in maternal-child health.

## Supporting information

S1 Appendix Manuscript COREQ checklist. (DOCX)

S2 Appendix Questionnaire for male partner focus group discussion. (DOCX)
