# Supplementary material for: Access to maternal-child health and HIV services for women in North-Central Nigeria: A qualitative exploration of the male partner perspective
Source: PLoS One. 2020 Dec 10;15(12):e0243611. doi: 10.1371/journal.pone.0243611 (PMC7728451; doi:10.1371/journal.pone.0243611)
Supplement: S1 Appendix — (DOCX) [file pone.0243611.s001.docx]

**Consolidated criteria for reporting qualitative studies (COREQ) checklist for:**

**“Access to Maternal-Child Health and HIV Services for Women in North-Central Nigeria: A Qualitative Exploration of the Male Partner Perspective”**

| **No.** | **Item** | **Guide Questions/Description** | **Response (and Location in Manuscript where applicable)** |
| --- | --- | --- | --- |
| **Domain 1: Research Team and Reflexivity** | | | |
| Personal Characteristics | | | |
| 1 | Interviewer/facilitator | Which author/s conducted the interview or focus group? | LJC (male), NASA and MAM (female), and three other male facilitators conducted the focus groups (Methods) |
| 2 | Credentials | What were the researcher’s credentials? E.g. PhD, MD | MAM (RN-MPH), NASA (MD), NT (MD), MHA (MD, MPH, DrPH), LJC (PhD) |
| 3 | Occupation | What was their occupation at the time of the study? | MAM, Research Associate  NASA, Senior Technical Advisor Pediatric HIV, and Principal Investigator  NT, Program Officer, Pediatric HIV  MHA, Preventive Medicine Practitioner and HIV Researcher  LJC, Social Science Professor |
| 4 | Gender | Was the researcher male or female? | Female authors: MAM, TN, NASA. Male authors: MHA and LJC. Overall, data analysis team comprised 2 females and 4 males including MAM, NASA and LJC. (Methods- Transcription and Data Analysis) |
| 5 | Experience and training | What experience or training did the researcher have? | MAM (Trained; plus 3 years of conducting and analyzing qualitative studies and surveys in study setting)  TN: (Trained; plus 5 years of conducting and analyzing studies and surveys in study setting)  LJC (over 25 years’ experience in teaching, conducting, analyzing and publishing qualitative studies)  NASA (Trained; plus 4 years of conducting and analyzing qualitative studies in study setting and over 10 years of research experience) |
| **Relationship with participants** | | | |
| 6 | Relationship established | Was a relationship established prior to study commencement? | No, there was no established relationship with participants prior to study commencement beyond recruitment activities (Methods- Study Population and Recruitment Procedures) |
| 7 | Participant knowledge of the interviewer | What did the participants know about the researcher? e.g. personal goals, reasons for doing the research | During orientation to study and consent process, researchers introduced themselves, stating where they worked (with an NGO/university and not for the health facility or government), and reasons for doing the research, namely, to improve the quality of health services to women and children living with, or affected by HIV. |
| 8 | Interviewer characteristics | What characteristics were reported about the interviewer/facilitator? Eg Bias, assumptions, reasons and interests in the research topic | Characteristics of interviewers and objectives are presented above and/or in the manuscript. The Social Scientist, LJC, is male and interested in the psychology and social determinants of health and disease among populations of African descent. The three other male facilitators of the FGDs in this manuscript were all Nigerian and multilingual, and each had between 2 and 20 years of experience conducting qualitative research in Nigeria and internationally. The Principal Investigator, NASA is a female paediatric infectious disease specialist interested in the prevention and treatment of HIV among African children. MAM is a female nurse, Nigerian, multilingual, familiar with the religious and cultural context of study setting and interested in health and socio-economic inequalities disproportionately affecting women and children. |
| **Domain 2: Study Design** | | | |
| Theoretical Framework | | | |
| 9 | Methodological orientation and Theory | What methodological orientation was stated to underpin the study? e.g. grounded theory, discourse analysis, ethnography, phenomenology, content analysis | Grounded theory with thematic analysis (Methods-Transcription and Data Analysis) |
| Participant selection | | | |
| 10 | Sampling | How were participants selected? e.g. purposive, convenience, consecutive, snowball | Purposive sampling for all participants. (Methods- Study Population and Recruitment Procedures) |
| 11 | Method of approach | How were participants approached? e.g. face-to-face, telephone, mail, email | First by healthcare workers and community gatekeepers (through phone calls and in person) to ascertain interest, then those interested were approached by research team in person. (Methods-Study Population and Recruitment Procedures) |
| 12 | Sample size | How many participants were in the study? | A total of 30 participants in 3 FGDs (Methods – Study Population and Recruitment Procedures) |
| 13 | Non-participation | How many people refused to participate or dropped out? Reasons? | All 30 participants who indicated interest showed up for the FGDs (Methods – Study Population and Recruitment Procedures) |
| **Setting** | | | |
| 14 | Setting of data collection | Where was the data collected? e.g. home, clinic, workplace | The 3-male partner FGDs were conducted at two Primary Healthcare Centers (one semi-rural and one rural), and one urban community (Methods-Study Design and Setting). |
| 15 | Presence of non-participants | Was anyone else present besides the participants and researchers? | No one else was present besides participants, facilitators, and observers. (Methods- Data Collection) |
| 16 | Description of sample | What are the important characteristics of the sample? e.g. demographic data, date | Study participants were all married. HIV status was not a criterion for inclusion (Methods – Study Population and Recruitment Procedures). Rest of study characteristics are in Table 1. |
| **Data collection** | | | |
| 17 | Interview guide | Were questions, prompts, guides provided by the authors? Was it pilot tested? | FGDs were guided by a semi-structured focus group guide, which is attached in Appendix. (Methods- Data Collection). The guide was not pilot-tested. |
| 18 | Repeat interviews | Were repeat interviews carried out? If yes, how many? | No, repeat interviews of participants were not conducted. |
| 19 | Audio/visual recording | Did the research use audio or visual recording to collect the data? | FGDs were audio-recorded, and later transcribed. There were no visual recordings. (Methods – Data Collection). |
| 20 | Field notes | Were field notes made during and/or after the interview or focus group? | Yes, an observer made field notes during the FGDs to augment data analysis and interpretation. (Methods- Data Collection) |
| 21 | Duration | What was the duration of the interviews or focus group? | Each FGD took 1 ½ to 2 hrs long. (Methods- Data Collection). |
| 22 | Data saturation | Was data saturation discussed? | While we may have reached thematic saturation for larger formative study (11 FGDs and 31 KIIs), we may not have necessarily reached thematic saturation for male partner-specific viewpoints due to the limited number of FGDs (3) conducted among male partners. (Study Limitations) |
| 23 | Transcripts returned | Were transcripts returned to participants for comment and/or correction? | No. Many of the participants could either not read or write or were not literate enough to be able to read and understand the transcripts regardless of whether they were written in English or local language. A verbal member check was not performed either, however the diverse and context-experienced facilitators and analyst team and our iterative analysis approach provided robust interpretation. |
| **Domain 3: Analysis and Findings** | | | |
| **Data analysis** | | | |
| 24 | Number of data coders | How many data coders coded the data? | Overall analysis was manually performed by a panel of six paired researchers, which included the FGD facilitators. (Methods-Transcription and Data Analysis) |
| 25 | Description of the coding tree | Did authors provide a description of the coding tree? | In initial coding, identified code words were categorized in hierarchical fashion into a coding tree, with parent (main) codes (“access”, “norms”, “support”) under which related subcategory codes were arranged. Ultimately, categories were combined or expanded to represent emergent themes. (Methods-Transcription and Data Analysis) |
| 26 | Derivation of themes | Were themes identified in advance or derived from the data? | We used the Grounded Theory approach to derive themes from the data. (Methods-Transcription and Data Analysis) |
| 27 | Software | What software, if applicable, was used to manage the data? | No specialized qualitative software was used to manage the data. |
| 28 | Participant checking | Did participants provide feedback on the findings? | No. Member checks were not performed. |
| **Reporting** | | | |
| 29 | Quotations presented | Were participant quotations presented to illustrate the themes / findings? Was each quotation identified? e.g. participant number | Participant quotations are presented and identified by participant group and number (Throughout Results section). |
| 30 | Data and findings consistent | Was there consistency between the data presented and the findings? | Yes, discussion on findings was written to align with Results/data presented |
| 31 | Clarity of major themes | Were major themes clearly presented in the findings? | Yes, the three major themes (Gender Power Dynamics; male partner preferences and options; and Care and Support) are clearly presented in the Results section and in Figure 1. |
| 32 | Clarity of minor themes | Is there a description of diverse cases or discussion of minor themes? | Yes, minor themes and/or divergent cases were discussed under each of the major themes (Results). |
